# The Effect of Different Output Powers of Blue Diode Laser along with Curcumin and Riboflavin against *Streptococcus mutans* around Orthodontic Brackets: An In Vitro Study

**DOI:** 10.3390/biomedicines11082248

**Published:** 2023-08-10

**Authors:** Edris Pordel, Trife Ghasemi, Shima Afrasiabi, Stefano Benedicenti, Antonio Signore, Nasim Chiniforush

**Affiliations:** 1 Department of Pediatric Dentistry, Dental School, Sabzevar University of Medical Sciences, Sabzevar 9613875389, Iran; edrispordel@yahoo.com; 2 Independent Researcher, Mashhad 9613875389, Iran; trifetrife@yahoo.com; 3 Laser Research Center of Dentistry, Dentistry Research Institute, Tehran University of Medical Sciences, Tehran 1441987566, Iran; 4 Department of Surgical Sciences and Integrated Diagnostics, University of Genoa, Viale Benedetto XV, 6, 16132 Genoa, Italy; benedicenti@unige.it; 5 Therapeutic Dentistry Department, Institute of Dentistry, I.M. Sechenov First Moscow State Medical University, Trubetskaya Str. 8, b. 2, 119992 Moscow, Russia; dr.signore@icloud.com

**Keywords:** antimicrobial photodynamic therapy, blue diode laser, curcumin, riboflavin, orthodontic brackets, dental caries

## Abstract

Objectives: The aim of the present study was to determine the effects of antimicrobial photodynamic therapy (aPDT) using the blue diode laser (BDL) with different output powers and the photosensitizers riboflavin and curcumin on reducing the number of *Streptococcus mutans* around orthodontic brackets. Materials and methods: A total of 36 orthodontic brackets were contaminated with *S. mutans* and randomly assigned to 12 groups as follows: control, riboflavin alone, riboflavin + BDL with an output power of 200, 300, 400, or 500 mW, and curcumin alone, curcumin + BDL with an output power of 200, 300, 400, or 500 mW, and 0.2% chlorhexidine (CHX-positive control). Orthodontic brackets were irradiated with a BDL (wavelength 445 nm) at a power density of 0.4–1.0 W/cm^2^ for 30 s. All orthodontic brackets were examined under a stereomicroscope at 10× magnification. Mean colony-forming units (CFUs)/mL were measured before and after treatment. A one-way analysis of variance with Tukey’s post hoc test was performed to compare CFU/mL between groups. Results: CHX and curcumin plus BDL with an output power of 500 mW had the highest reduction in *S. mutans* colony numbers (*p* < 0.001). The curcumin groups were more effective than the riboflavin groups. Riboflavin alone and riboflavin + BDL with an output power of 200 mW showed no significant difference from the control group (*p* = 0.99 and 0.74, respectively). Conclusion: Our results suggest that aPDT using curcumin as a photosensitizer plus BDL with an output power of 500 mW and a power density of 1.0 W/cm^2^ at a wavelength of 445 nm can effectively reduce colonies of *S. mutans* around stainless steel brackets.

## 1. Introduction

The goal of orthodontic treatment for misaligned teeth is to improve the appearance of the mouth and jaw region and to make chewing easier by realigning the teeth. The side effects of orthodontic therapy include periodontal disease, root resorption, temporomandibular joint disorders, dental caries, and enamel degradation [1]. Dental caries is a severe, contagious, bacterial infection with complex pathophysiology. Cariogenic bacteria, fermentable carbohydrates, a susceptible tooth, the host, and time are the main factors contributing to the development of this disease [2]. It is a major economic burden and public health problem throughout the world [3]. *Streptococcus mutans* is the main cause of dental caries due to its ability to synthesize glucans, form biofilms, and produce acid. Treatment methods commonly used for caries prevention include mechanical devices, antibacterial agents, fluoride therapy, and immunizations [4,5]. Antimicrobial photodynamic therapy (aPDT) is a strategy to suppress cariogenic bacteria and prevent periodontal disease. This procedure is safe in the long term as it has no genotoxic and mutagenic consequences. The advantages of this technique include rapid reduction in bacteria, no invasion and damage to surrounding tissues, accessibility to sites with complicated architecture, low risk of bacteremia, and excellent repeatability [6]. The aPDT method includes a photosensitizer, oxygen, and light. In the presence of oxygen, the photosensitizer generates reactive oxygen species (ROS) and other free radicals upon exposure to light, which lead to irreversible destruction of cellular components, alter signal transduction pathways, and disrupt metabolic processes, ultimately resulting in cell death [7].

Recently, natural photosensitizers such as riboflavin and curcumin have come into focus. Riboflavin, a water-soluble vitamin, and curcumin possess biocompatibility, nontoxicity, and the ability to produce ROS that have recently been investigated for their potential antibacterial effects [8]. Riboflavin is an efficient photosensitizer that causes oxidative damage when activated by visible light, especially blue light [6]. Kamran et al. used a blue LED with a light intensity of 2000 mW/cm^2^ and a wavelength range of 450 nm and fluence of 95 J/cm^2^ to investigate the antimicrobial potential of riboflavin-mediated aPDT. They showed that the method significantly reduced the amount of *S. mutans* around metallic brackets [9]. Comeau et al. showed that riboflavin with blue LED groups caused a lower number of *S. mutans* colony-forming unit (CFU)/mL compared to the control group [10]. The highest absorption range for riboflavin is at wavelengths of 445, 336, and 270 nm [11].

For curcumin, it is around 300–500 nm with maximum absorption at a wavelength of 425 nm [12]. Curcumin is a hydrophobic photosensitizer that is soluble in polar solvents such as dimethyl sulfoxide (DMSO), acetonitrile, methanol, oils, etc. [13]. Curcumin adheres to the lipid membrane and bacterial proteins and damages the bacterial membrane by various mechanisms. Due to its ability to absorb blue light and generate ROS, it has shown significant potential as a photosensitizer [14]. Previously, Azizi et al. demonstrated in an experimental in vitro study that curcumin-mediated aPDT using 460 nm/100 mW laser (60 s) can considerably reduce *S. mutans* colonies [15]. Lee et al. used a 405 nm light-emitting diode (LED) with a power density of 84.5 mW for 5 min at an energy density of 25.3 J/cm^2^ and curcumin as a photosensitizer. They showed that under the above conditions, the viability of *S. mutans* was significantly reduced [16]. Tonon et al. showed that the group irradiated with blue LED in a power density of 240.1 mW/cm^2^ and in the presence of curcumin achieved a significantly lower number of viable *S. mutans* compared to the control group [17]. The aim of the present study was to determine the effects of different output powers of blue diode laser (BDL) with the photosensitizers riboflavin and curcumin on reducing the number of *S. mutans* around orthodontic brackets. The null hypothesis was tested that there is no difference between different output powers of BDL during aPDT against *S. mutans* around orthodontic brackets.

## 2. Materials and Methods

### 2.1. Sample Size

According to the conditions of α = 0.05, β = 0.2, standard deviation = 8.66, and effect size = 0.45, the sample size was calculated using the one-way analysis of variance (ANOVA) in IBM SPSS Statistics 25.0 (Armonk, NY, USA) and was set to 3 orthodontic brackets in each group.

### 2.2. Sample Preparation

The study protocol was approved by the Ethics Committee of the Tehran University of Medical Sciences (IR. TUMS. DENTISTRY.REC. 1400. 187). A total of 36 healthy human premolar teeth with no carious were bonded to 0.022, stainless steel brackets (TSHdental, Tehran, Iran). The teeth were extracted for orthodontic reasons. A stereomicroscope (SMZ800, Nikon, Tokyo, Japan) was used to examine the orthodontic brackets at a magnification of 10×. The orthodontic brackets displayed normal architecture and healthy enamel on the buccal surface, free of any cracks, fractures, or restorations in the enamel. No pretreatments for bleaching or aPDT were recorded in the patient records. A periodontal scaler was used to remove the remaining soft tissue around the orthodontic brackets, and all dentin surfaces were polished for 15 s with a low-speed handpiece (Coxo, Guangzhou, China), rubber cups (Microdont, São Paulo, Brazil), and pumice paste (Cina, Tehran, Iran) before being thoroughly washed under tap water. Orthodontic brackets were cleaned with a 0.5% (*w*/*v*) chloramine T (MF aqua, Tehran, Iran) solution for one week at 3 °C and then stored in saline until the start of the experiment.

### 2.3. Microbial Suspension

*S. mutans* (IBRC-M 10,682) was provided by the Iranian Biological Resource Centre (Tehran, Iran) for this study. Bacteria was inoculated in 10 mL of brain heart infusion (BHI) broth (Ibresco, Tehran, Iran) and were incubated under an aerobic atmosphere at 37 °C overnight. A 1.5 × 10^8^ CFU/mL bacterial suspension equivalent to 0.5 McFarland was prepared. A spectrophotometer (Spectroshade Micro, MHT, Verona, Italy) was used for verification, indicating a value between 0.8 and 0.13 at a wavelength of 625 nm. Orthodontic brackets were placed in a 24-well microplate and contaminated with 1 mL of *S. mutans* suspension (10^6^ CFU/mL). Subsequently, samples were incubated at 37 °C for 72 h for biofilm formation.

### 2.4. Experimental Groups

The current study included 12 groups with three orthodontic brackets, including:

1. Control group;
2. Riboflavin alone;
3. Riboflavin + BDL (output power = 200 mW);
4. Riboflavin + BDL (output power = 300 mW);
5. Riboflavin + BDL (output power = 400 mW);
6. Riboflavin + BDL (output power = 500 mW);
7. Curcumin alone;
8. Curcumin + BDL (output power = 200 mW);
9. Curcumin + BDL (output power = 300 mW);
10. Curcumin + BDL (output power = 400 mW);
11. Curcumin + BDL (output power = 500 mW);
12. 0.2% chlorhexidine mouthwash (CHX; Vi-One, Tabriz, Iran) as positive control.

### 2.5. Photosensitizers, Light Source, and aPDT

Curcumin (UltraCur, weber medical, Lauenförde, Germany) was diluted with <1% DMSO (Sigma-Aldrich, St. Louis, MO, USA) in phosphate-buffered solution to reach a final concentration of 40 µM. Riboflavin (Harman Finochem Ltd., Mumbai, India) was dissolved in 0.9% NaCl to reach a final concentration of 100 µM. Before starting the experiment, both photosensitizers were stored in a dark room. One milliliter of the riboflavin or curcumin solution was applied to the contaminated orthodontic brackets. The samples were illuminated at room temperature using a BDL (Wiser 3, Doctor Smile, Brendola, Italy) with a wavelength of 445 nm and output powers of 200, 300, 400, and 500 mW for 30 s. The tip diameter was 8 mm, the surface area was considered as 0.5 cm^2^, and the distance between the tip and the samples was 1 mm. After treatment, each orthodontic bracket was inserted in microtube containing 1 mL BHI broth and vortexed for 2 min. Then, 10 μL from each solution was diluted (10^−1^–10^−5^) and transferred to BHI agar (Ibresco, Tehran, Iran) plates. The plates were incubated under aerobic incubation at 37 °C for 48 h. The number of CFU/mL was determined according to the previously described method of Miles and Misra [18].

### 2.6. Statistical Analysis

IBM SPSS 25.0 statistics was used for all calculations. Descriptive statistics were obtained for each group, including mean, standard deviation, and minimum and maximum values. Mean and log_10_ CFU/mL was compared between groups using the ANOVA and a post hoc Tukey’s test. The significance threshold was set at *p* < 0.05.

## 3. Results

**Figure 1:**
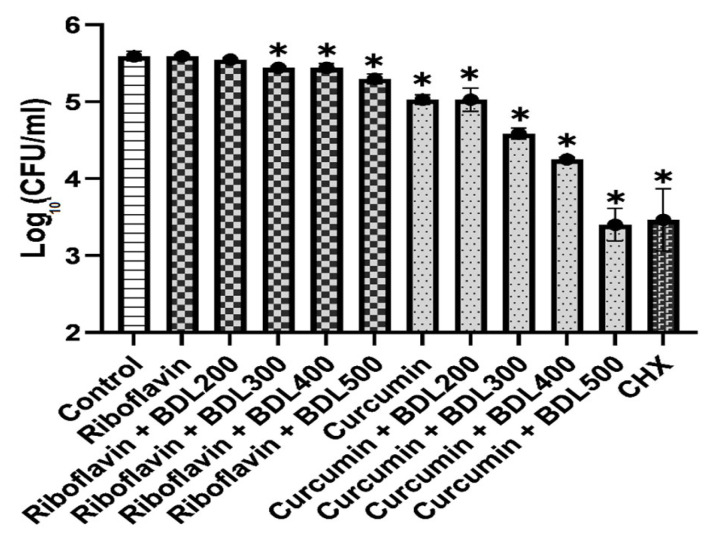
Log_10_ CFU/mL of *Streptococcus mutans* of the study groups. * Significantly different compared to control group. BDL: blue diode laser; CHX: chlorhexidine.

As shown in Figure 1, study groups with higher output power caused less viable bacteria around orthodontic brackets. The details of the analysis between groups and the results of Tukey’s tests are shown in Table 1. Post hoc analysis of the log_10_ CFU/mL showed that the riboflavin + BDL with an output power of 200 or 300 mW has a significant difference from the riboflavin + BDL with an output power of 500 mW (*p* < 0.001). Likewise, a significant difference was observed between curcumin + BDL with an output power of 200 mW and curcumin + BDL with an output power of 400 or 500 mW (*p* = 0.01 and <0.001, respectively). However, no significant difference was found between BDL with an output power of 200 mW and 300 mW, as well as BDL with an output power of 400 mW and 500 mW, in any of the photosensitizer groups. Moreover, only riboflavin alone and riboflavin + BDL with an output power of 200 mW failed to significantly eliminate the amount of bacteria around orthodontic brackets. In addition, all curcumin groups were significantly more capable of reducing bacteria around orthodontic brackets than riboflavin groups.

## 4. Discussion

Orthodontic brackets and ligature wires that have rough and viable areas in and around them are an important source of plaque accumulation that puts patients at risk for periodontal disease [19]. *S. mutans* is able to adhere to types of orthodontic brackets and form biofilms [20]. The model of the bracket and the reactions of the components of the method, including brackets, photosensitizers, and light, may affect the growth of bacteria and the effectiveness of the method [19]. However, a previous study has found no significant difference in *S. mutans* adhesion between different types of orthodontic brackets, including stainless steel, metal, composite, ceramic, and plastic brackets [21].

According to the findings of this study, the null hypothesis can be rejected. This study showed that curcumin + BDL with an output power of 500 mW was the most effective group, being as effective as CXH. CHX is a popular antimicrobial agent in dentistry that effectively eliminates microbes. It is the gold standard for the destruction of microbial biofilms [22]. However, it has some disadvantages, including taste impairment and discoloration of teeth and mucous membranes [23]. All groups receiving curcumin as a photosensitizer were more effective than groups receiving riboflavin, except for the curcumin alone, which showed no significant difference from riboflavin + BDL with an output power of 500 mW. The riboflavin alone and riboflavin + BDL with an output power of 200 mW groups showed no significant difference from the control group. However, neither group was able to completely kill all microorganisms.

One of the main reasons for the conflicting results within the groups is due to the properties of the photosensitizers. In this experiment, the antibacterial activity of aPDT with curcumin or riboflavin photosensitizers on *S. mutans* biofilms was evaluated by calculating CFU/mL. Araujo and coworkers studied the efficacy of curcumin in combination with blue LED on salivary bacteria of 13 adults and found a considerable decrease in bacterial viability [24]. Paschoal et al. also showed that the group receiving curcumin plus blue LED had a 70% greater reduction in bacterial counts than the control group under certain experimental conditions [25]. Our findings in the present study are consistent with those of previous studies. Kamran et al. [9] clearly demonstrated that riboflavin at a total concentration of 0.5% effectively reduced the viability of *Streptococcus sanguinis* and *S. mutans* biofilms around fixed orthodontic brackets using blue LED light with a power density of 2000 mW/cm^2^, a wavelength range of 450 ± 65, and an energy density of 95 J/cm^2^. The biofilms on the orthodontic brackets of the group receiving riboflavin alone were mostly viable. They showed that blue LED with riboflavin is as effective as CHX, but we did not observe such results. An important difference between Kamran et al. and our study is that they used metal brackets, whereas we used stainless steel brackets, which may have affected the results. Another study investigated the efficacy of riboflavin-mediated aPDT using BDL with different output powers (200–500 mW). They showed that irradiation of bacterial cultures with a BDL in the presence of riboflavin as a photosensitizer resulted in a dose-dependent decrease in *Enterococcus faecalis* viability [26]. The results of our study showed that the antibacterial potential of aPDT method increased with increasing strength (BDL), with curcumin + BDL with an output power of 500 mW showing the highest efficacy among the study groups. In agreement with our study, this study showed a greater reduction in *E. faecalis* at higher power of BDL. However, BDL alone at 200 mW to 500 mW showed no significant difference between the different output powers [26]. Other studies have shown that BDL or blue LED with riboflavin as a photosensitizer reduces colony numbers of *S. mutans*, *E. faecalis*, *Staphylococcus aureus*, and *Escherichia coli* to varying degrees [9,26,27,28,29]. The differences in the results on the efficacy of aPDT with riboflavin may be due to different bacterial strains, the site of the procedure, the concentration of the photosensitizer substance, the duration of the light source, and different laser parameters. However, Etemadi et al. have shown in a systematic review that there is no study reporting that riboflavin-mediated aPDT cannot reduce the number of bacterial colonies [30]. While we observed a large difference between riboflavin-containing groups and CHX, Morelato et al. recently showed that aPDT at 445 nm (Q power = 100 mW, 100 Hz, 124.34 W/cm^2^, 1.24 J/cm^2^) with a 0.1% riboflavin dye is as effective as 0.2% CHX. This contradiction may be explained by the microbial species used (*S. aureus* and *Candida albicans*) or the procedure [29].

A comparison of twelve groups and the different BDL irradiation powers were strengths of our study. Undoubtedly, this study has some limitations. The evaluation of antimicrobial capacity at a single time point is not sufficient to justify the potential antibacterial efficacy. Moreover, the present results could only be studied on a monomicrobial biofilm. Future studies should test the effect of the combination of BDL and curcumin/riboflavin on multiple microorganisms and determine the significance of curcumin/riboflavin concentration and BDL parameters for each strain tested. In addition, this study was designed as an in vitro study, which limits the transferability of the results to in vivo conditions and clinical setting. In vitro studies cannot determine environmental factors such as restricted accessibility, plaque formation, salivary flow, immune system influence, etc. Clinical studies are needed to obtain more reliable results. Another limitation of the study could be the relatively small sample size. Our results encourage further investigation of the in vitro and in vivo effects of novel disinfection therapies using curcumin/riboflavin and BDL. In addition, the shear bond strength of our method should be investigated in future studies.

## 5. Conclusions

This study showed that curcumin-mediated aPDT was more effective than riboflavin-mediated aPDT. The higher output powers were more effective in the reduction in *S. mutans* CFU/mL. The microbial decontamination potential of curcumin/riboflavin with BDL makes it possible to expand the scope of this device.

## Figures and Tables

**Table 1:** Comparison between groups based on mean colony count of *Streptococcus mutans* after intervention.

Group 1	Group 2	*p*-Value
Riboflavin	Riboflavin + BDL 200 mW	0.85
	Riboflavin + BDL 300 mW	0.03 *
	Riboflavin + BDL 400 mW	0.00 *
	Riboflavin + BDL 500 mW	0.00 *
	CHX	0.00 *
Riboflavin + BDL 200 mW	Riboflavin + BDL 300 mW	0.67
	Riboflavin + BDL 400 mW	0.07
	Riboflavin + BDL 500 mW	0.00 *
	CHX	0.00 *
Riboflavin + BDL 300 mW	Riboflavin + BDL 400 mW	0.95
	Riboflavin + BDL 500 mW	0.00 *
	CHX	0.00 *
Riboflavin + BDL 400 mW	Riboflavin + BDL 500 mW	0.06
	CHX	0.00 *
Riboflavin + BDL 500 mW	CHX	0.00 *
Curcumin	Curcumin + BDL 200 mW	0.00 *
	Curcumin + BDL 300 mW	0.00 *
	Curcumin + BDL 400 mW	0.00 *
	Curcumin + BDL 500 mW	0.00 *
	CHX *	0.00 *
Curcumin + BDL 200 mW	Curcumin + BDL 300 mW	0.10
	Curcumin + BDL 400 mW	0.01 *
	Curcumin + BDL 500 mW	0.00 *
	CHX	0.00 *
Curcumin + BDL 300 mW	Curcumin + BDL 400 mW	0.99
	Curcumin + BDL 500 mW	0.87
	CHX	0.89
Curcumin + BDL 400 mW	Curcumin + BDL 500 mW	1.00
	CHX	1.00
Curcumin + BDL 500 mW	CHX	1.00

Abbreviation: BDL: blue diode laser, CXH: chlorhexidine. * Significantly different compared to other group.

## Data Availability

Not applicable.

## References

[1] Wishney M. (2017). Potential risks of orthodontic therapy: A critical review and conceptual framework. Aust. Dent. J..

[2] Ahirwar S.S., Gupta M., Snehi S.K. (2019). Dental caries and lactobacillus: Role and ecology in the oral cavity. Int. J. Pharm. Sci. Res..

[3] Qin X., Zi H., Zeng X. (2022). Changes in the global burden of untreated dental caries from 1990 to 2019: A systematic analysis for the Global Burden of Disease study. Heliyon.

[4] Abrar E., Naseem M., Baig Q.A., Vohra F., Maawadh A.M., Almohareb T., AlRifaiy M.Q., Abduljabbar T. (2020). Antimicrobial efficacy of silver diamine fluoride in comparison to photodynamic therapy and chlorhexidine on canal disinfection and bond strength to radicular dentin. Photodiagn. Photodyn. Ther..

[5] Ahmadi H., Ebrahimi A., Ahmadi F. (2021). Antibiotic therapy in dentistry. Int. J. Dent..

[6] Cieplik F., Deng D., Crielaard W., Buchalla W., Hellwig E., Al-Ahmad A., Maisch T. (2018). Antimicrobial photodynamic therapy—What we know and what we don’t. Crit. Rev. Microbiol..

[7] Moro G.G., Massat N.C., Grandizoli D.R.P., Junior A.E., Degasperi G.R., Fontana C.E., Pinheiro S.L. (2021). Effect of cetrimide 2% with and without photodynamic therapy to reduce *Streptococcus mutans* burden in dentinal carious lesions. Lasers Med. Sci..

[8] Anand P., Kunnumakkara A.B., Newman R.A., Aggarwal B.B. (2007). Bioavailability of curcumin: Problems and promises. Mol. Pharm..

[9] Kamran M.A., Qasim M., Udeabor S.E., Hameed M.S., Mannakandath M.L., Alshahrani I. (2021). Impact of riboflavin mediated photodynamic disinfection around fixed orthodontic system infected with oral bacteria. Photodiagn. Photodyn. Ther..

[10] Comeau P., Burgess J., Qomi N.R., Lee A., Manso A. (2022). The antimicrobial, physical, and chemical properties of a riboflavin-loaded dental resin intended for antimicrobial photodynamic therapy. Photodiagn. Photodyn. Ther..

[11] Fawzy A.S., Nitisusanta L.I., Iqbal K., Daood U., Neo J. (2012). Riboflavin as a dentin crosslinking agent: Ultraviolet A versus blue light. Dent. Mater..

[12] Subhan M.A., Alam K., Rahaman M.S., Rahman M.A., Awal R. (2013). Synthesis and characterization of metal complexes containing curcumin (C21H20O6) and study of their anti-microbial activities and DNA-binding properties. J. Sci. Res..

[13] Priyadarsini K.I. (2014). The chemistry of curcumin: From extraction to therapeutic agent. Molecules.

[14] Leite D.P.V., Paolillo F.R., Parmesano T.N., Fontana C.R., Bagnato V.S. (2014). Effects of photodynamic therapy with blue light and curcumin as mouth rinse for oral disinfection: A randomized controlled trial. Photomed. Laser Surg..

[15] Azizi A., Shohrati P., Goudarzi M., Lawaf S., Rahimi A. (2019). Comparison of the effect of photodynamic therapy with curcumin and methylene Blue on streptococcus mutans bacterial colonies. Photodiagn. Photodyn. Ther..

[16] Lee H.J., Kang S.M., Jeong S.H., Chung K.H., Kim B.I. (2017). Antibacterial photodynamic therapy with curcumin and Curcuma xanthorrhiza extract against Streptococcus mutans. Photodiagn. Photodyn. Ther..

[17] Tonon C.C., Paschoal M.A., Correia M., Spolidório D.M., Bagnato V.S., Giusti J.S., Santos-Pinto L. (2015). Comparative effects of photodynamic therapy mediated by curcumin on standard and clinical isolate of *Streptococcus mutans*. J. Contemp. Dent. Pract..

[18] Miles A.A., Misra S.S., Irwin J.O. (2009). The estimation of the bactericidal power of the blood. J. Hyg..

[19] Antoun J.S., Mei L., Gibbs K., Farella M. (2017). Effect of orthodontic treatment on the periodontal tissues. Periodontol. 2000.

[20] Papaioannou W., Panagopoulos A., Koletsi-Kounari H., Kontou E., Makou M. (2012). Adhesion of Porphyromonas gingivalis and biofilm formation on different types of orthodontic brackets. Int. J. Dent..

[21] Papaioannou W., Gizani S., Nassika M., Kontou E., Nakou M. (2007). Adhesion of Streptococcus mutans to Different Types of Brackets. Angle Orthod..

[22] Balagopal S., Arjunkumar R. (2013). Chlorhexidine: The Gold Standard Antiplaque Agent. J. Pharm. Sci..

[23] Polizzi E., TetÃ G., Bova F., Pantaleo G., Gastaldi G., CapparÃ P., Gherlone E. (2019). Antibacterial properties and side effects of chlorhexidine-based mouthwashes. A prospective, randomized clinical study. J. Osseointegr..

[24] Araújo N.C., Fontana C.R., Gerbi M.E., Bagnato V.S. (2012). Overall-mouth disinfection by photodynamic therapy using curcumin. Photomed. Laser Surg..

[25] Paschoal M.A., Tonon C.C., Spolidório D.M., Bagnato V.S., Giusti J.S., Santos-Pinto L. (2013). Photodynamic potential of curcumin and blue LED against *Streptococcus mutans* in a planktonic culture. Photodiagn. Photodyn. Ther..

[26] Afrasiabi S., Chiniforush N. (2023). Antibacterial potential of riboflavin mediated blue diode laser photodynamic inactivation against Enterococcus faecalis: A laboratory investigation. Photodiagn. Photodyn. Ther..

[27] Nielsen H.K., Garcia J., Væth M., Schlafer S. (2015). Comparison of riboflavin and toluidine Blue O as photosensitizers for photoactivated disinfection on endodontic and periodontal pathogens in vitro. PLoS ONE.

[28] Banerjee S., Ghosh D., Vishakha K., Das S., Mondal S., Ganguli A. (2020). Photodynamic antimicrobial chemotherapy (PACT) using riboflavin inhibits the mono and dual species biofilm produced by antibiotic resistant *Staphylococcus aureus* and *Escherichia coli*. Photodiagn. Photodyn. Ther..

[29] Morelato L., Budimir A., Smojver I., Katalinić I., Vuletić M., Ajanović M., Gabrić D. (2022). A novel technique for disinfection treatment of contaminated dental implant surface using 0.1% riboflavin and 445 nm diode laser-an in vitro study. Bioengineering.

[30] Etemadi A., Hamidain M., Parker S., Chiniforush N. (2021). Blue light photodynamic therapy with curcumin and riboflavin in the management of periodontitis: A Systematic Review. J. Lasers Med. Sci..

